# Microbial micronutrient sharing, gut redox balance and keystone taxa as a basis for a new perspective to solutions targeting health from the gut

**DOI:** 10.1080/19490976.2025.2477816

**Published:** 2025-03-16

**Authors:** Robert E. Steinert, Ateequr Rehman, Mehdi Sadaghian Sadabad, Alessio Milanese, Jonas Wittwer-Schegg, Jeremy P. Burton, Anneleen Spooren

**Affiliations:** a Health, Nutrition & Care (HNC), Dsm-Firmenich, Kaiseraugst, Switzerland; b Department of Surgery and Transplantation, University Hospital Zurich (USZ) and University of Zurich (UZH), Zürich, Switzerland; c Data Science, Science & Research, Dsm-Firmenich, Delft, Netherlands; d Department of Microbiology and Immunology, The University of Western Ontario, London, Canada

**Keywords:** Microbiome, pantryome, crossfeeding, biotics, humans, gastrointestinal, host health

## Abstract

In health, the gut microbiome functions as a stable ecosystem maintaining overall balance and ensuring its own survival against environmental stressors through complex microbial interaction. This balance and protection from stressors is maintained through interactions both within the bacterial ecosystem as well as with its host. As a consequence, the gut microbiome plays a critical role in various physiological processes including maintaining the structure and function of the gut barrier, educating the gut immune system, and modulating the gut motor, digestive/absorptive, as well as neuroendocrine system all of which are crucial for human health and disease pathogenesis. Pre- and probiotics, widely available and clinically established, offer various health benefits primarily by beneficially modulating the gut microbiome. However, their clinical outcomes can vary significantly due to differences in host physiology, diets, individual microbiome compositions, and other environmental factors. This perspective paper highlights emerging scientific insights into the importance of microbial micronutrient sharing, gut redox balance, keystone species, and the gut barrier in maintaining a diverse and functional microbial ecosystem, and their relevance to human health. We propose a novel approach that targets microbial ecosystems and keystone taxa performance by supplying microbial micronutrients in the form of colon-delivered vitamins, and precision prebiotics [e.g. human milk oligosaccharides (HMOs) or synthetic glycans] as components of precisely tailored ingredient combinations to optimize human health. Such a strategy may effectively support and stabilize microbial ecosystems, providing a more robust and consistent approach across various individuals and environmental conditions, thus, overcoming the limitations of current single biotic solutions.

## 1. Introduction

In humans, microbial communities form complex, balanced ecosystems at distinct body sites including nose, mouth, skin, vagina, and the gastrointestinal (GI) tract. The latter harbors the largest part of the human microbiome (>90%) containing about 10^14^ microorganisms of more than 500 different species. The numbers of bacteria generally increase going down the GI tract, ranging from 100 to 1000 per ml in the highly acidic environment of the stomach to about 10^5^ per ml in the upper small intestine and up to 10^12^ per ml in the anaerobic colon.^1^ The number of microorganisms in the large intestine is estimated to be equivalent to the number of human cells in the whole body^2^ while encoding a genetic repertoire that outnumbers human genes by at least two orders of magnitude.^3^ This high density of genetic information suggests a crucial role for host physiology despite the long-standing view of the colon as merely an organ that only re-absorbs water and electrolytes and processes undigested foods. In fact, recent advances in DNA sequencing and bioinformatics combined with functional studies in experimental rodents have revolutionized our understanding of gut microbiome–host interactions and emphasize the critical role of the gut microbiome in maintaining human health and contributing to the pathogenesis of many diseases.

**Figure 1.**
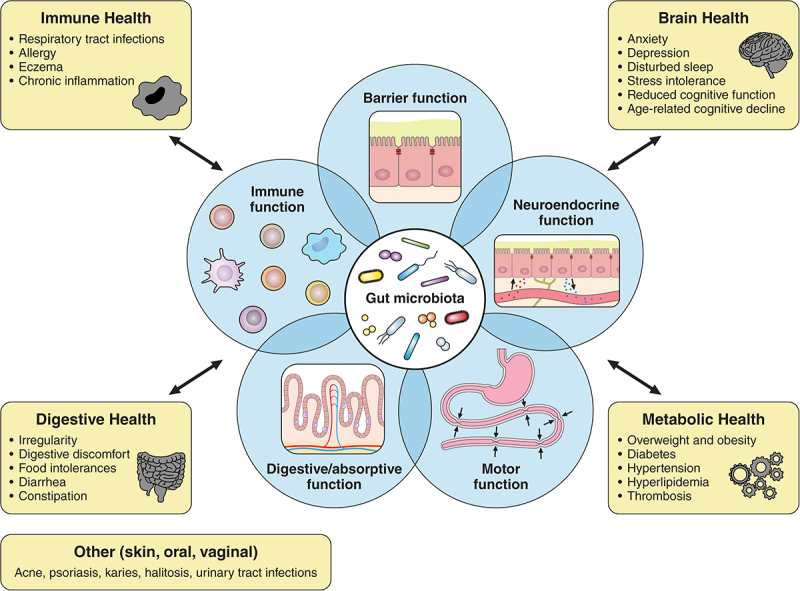
The gut microbiota and human health. Schematic illustration of how the gut microbiota interacts with the principal functions of the GI tract: digestion and absorption of food (1); gut motor function for the progression of food (2); gut neuroendocrine function to regulate metabolic responses (3); and gut barrier and immune function to protect against harmful pathogens, toxins, and antigens (4 and 5). These interactions play a critical role in overall host health, well-being, and disease pathogenesis. Many of these functions are interrelated and directly modulated by the gut microbiota. For instance, the production of short-chain fatty acids (SCFAs) stimulates the secretion of gastrointestinal hormones (secretin, motilin, glucagon-like peptide-1, peptide YY, etc.) by acting on G-protein coupled receptors, coordinating the secretion of digestive enzymes, intestinal transit, mucus production, postprandial blood glucose levels, appetite, and gut-brain axis communication, which links to emotional and cognitive performance. Altogether, this contributes to the main health benefits, including immune, digestive, brain, and metabolic health. Skin, oral, and vaginal health are also related, but to a lesser extent.

In health, the gut microbiome acts as a balanced ecosystem playing a critical role in various physiological processes. These include maintaining the structure and function of the gut barrier, educating the gut immune system, and modulating the gut motor, digestive/absorptive, as well as neuroendocrine system. Many of these functions are interconnected, for example, the digestion of dietary components by microbes leads to the production of butyrate and other short chain fatty acids (SCFA) which decreases luminal pH and reduces oxygen levels and thus protects against pH-sensitive or aerotolerant pathogen overgrowth.^4^ SCFA and other metabolic products also strengthen the gut barrier, modulate immune responses and impact gut neuroendocrine and motor function as well as nutrient absorption.^4–6^ Figure 1 depicts a schematic drawing of how the gut microbiome interacts with the basic physiological functions of the GI tract and how this relates to overall human health. Accumulating evidence suggests that perturbations of gut microbial balance and related GI functionality may drive infections, obesity, diabetes as well as various inflammatory, digestive, and neurological disorders suggesting a huge potential for interventions targeting the gut microbiome^7–9^

Pre- and probiotics are widely available on the market for a few decades now and are recognized for their various health benefits, primarily through their role in modulating the gut microbiome. They are defined either as live microorganisms that, when administered in adequate amounts, confer a health benefit on the host,^10^ or as substrates that are selectively utilized by host microorganisms, providing a health benefit.^11^ Most recently, also postbiotics became recognized and are defined as a preparation of inanimate microorganisms and/or their components that confers a health benefit on the host.^12^ Despite the fact that particularly pre and probiotics are clinically well established for a variety of health benefits such as to reduce antibiotic-associated diarrhea, to help manage digestive discomforts, or decrease risk or duration of common infections, including respiratory tract, gut and vaginal tract infections, they often can exhibit high variability in clinical outcomes.^13^ This variability arises due to differences in host physiology, diets, individual microbiome compositions, varying levels of product quality controls, and other environmental factors. Consequently, there is a growing need for next-generation gut health ingredients specifically designed to address and mitigate these disparities. Additionally, stability and precise dosing are critical considerations, as the living nature of probiotics and the high doses required for traditional prebiotics typically constrain their applicability in new product formulations or ingredient combinations.

In this perspective paper, we briefly highlight recent scientific insights into the importance of microbial micronutrient sharing, gut redox balance, keystone species, and the gut barrier in maintaining a diverse and functional microbial ecosystem and their relevance to host health. We further explore how to incorporate these emerging insights into novel approaches targeting microbiome health by supplying microbial micronutrients (e.g. in the form of colon-delivered vitamins) and precision prebiotics (e.g. in the form of human milk oligosaccharides (HMOs) or synthetic glycans) as components of precisely tailored ingredient combinations. Such a strategy may effectively support microbial ecosystems and broader gut functionility, thereby offering a more robust and consistent approach across various individuals and environmental conditions to benefit human health

## 2. Development of the gut microbiome from early life to adulthood

The first couple of months of life is a critical window for microbiome development. Several factors, such as mode of delivery, diet, environment, and the use of antibiotics, shape a child’s gut microbiota, which can have a profound impact on childhood development and lifelong health. For example, recent studies suggest that infants whose microbiome development is disrupted via cesarean section delivery, early antibiotic use, limited breastfeeding, or other factors are at greater risk for asthma and allergies, respiratory infections, inflammatory bowel disease (IBD), type 1 diabetes, and obesity.^14,15^ The assembly of the infant microbiome is first determined by maternal – infant exchanges of microbiota.^16^ Therefore, optimizing the maternal microbiome during pregnancy is likely part of a comprehensive approach to protect and promote the fetus’s health and provide the newborn with a specific microbial inoculum at birth.^14,17^ After birth, maternal breast milk promotes the colonization and maturation of the infant’s gut microbiome. Human milk contains a high concentration of indigestible glycans, known as HMOs which can act as growth substrates for beneficial *Bifidobacteria* to support the early founder strains of the infant microbiome.^18,19^ In addition, HMOs extert several microbiome-independent mechanism such as serving as decoy receptors to effectively block the attachment of pathogenic bacteria and directly interacting with various receptors.^20^ The infant microbiome evolves and diversifies further throughout life in response to whether an infant is breastfed, or formula-fed and which type of formula is used. The weaning period (i.e. the introduction of solid food at around 4–12 months) represents another important window of opportunity to positively impact the development of the microbiome as the bacterial community needs to adapt to digest dietary fibers. Studies linking low gut microbial diversity and the lack of specific bacteria to atopic dermatitis emphasize the first 18 month as a critical window period.^21^ Complete gut colonization then occurs within approximately 3 years of life and plays an essential role in further digestion, immunity and neuroendocrine pathway development.^22,23^ In cases where antibiotic treatment is necessary, biotic supplements (i.e. pre, pro, syn or postbiotics) have been shown to lessen the deleterious impact of antibiotics on the infant gut microbiome.^24,25^

In healthy adults, the gut microbiome is fully developed and designed to maintain overall balance while promoting its own survival against environmental stressors, with microorganisms engaging in complex interactions. Recent high-resolution studies examining microbiome composition before, during, and after antibiotic use at the individual gene x strain level demonstrate the remarkable adaptability or ‘fitness’ of gut microbial ecosystems.^26,27^ A healthy and fully functional ecosystem primarily aims to preserve its balance, with ecological diversification playing a crucial role in shaping the genetic structure of resident populations to defend against competition and external disruptors and stressors.^27,28^ Thus, intestinal bacterial ecosystems seem to carry an inherent ecological resilience helping to protect both, themselves and as a consequence their host’s health. This resilience seems to be driven by two main factors: a more diverse microbiome appears generally better at preserving its own balance;^29,30^ and 2) a highly collaborative and interdependent nature of microbial communities seems to play a key role in ecological resilience. In a healthy state, different species work together in a balanced and mutually beneficial way through mechanisms like crossfeeding of various microbial nutrients beyond SCFAs and other forms of metabolic cooperation to stabilize bacterial communities under varying environmental conditions.^31^ Understanding these mechanisms across all life stages, from infancy to adulthood to old age, while accounting for the variability in adult microbiome profiles shaped by factors such as genetics, diet, lifestyle, and environmental exposures^32–35^ will likely enable the design of better-tolerated and more precise interventions. These interventions could holistically target the functionality of microbiome networks rather than focusing solely on individual species or strains and, thus, allow to tap into the endogenous biochemical pathways that act to maintain bacterial homeostasis

The large diversity of the adult microbiome, however, presents a notable challenge. A previous comprehensive investigation involving over 1000 healthy individuals from diverse ancestral backgrounds living in shared environments provided interesting insights. It showed that genetic ancestry has minimal influence on gut microbiome composition. Instead, notable similarities were found in the microbiomes of unrelated individuals sharing the same household with over 20% of the differences in microbiome composition between individuals attributed to factors such as habitual diet, medication use, and anthropometric measurements.^36^ This is supported by findings from controlled-feeding studies in humans^35^ ^37^ For example, microbiome composition changed detectably within 24 h of initiating a high-fat/low-fiber *vs*. a low-fat/high-fiber diet ^37^ despite entrotype stability. Thus, defining a ´healthy´ microbiome at population levels will remain challenging,^38^ instead microbial dysbiosis is often used to link changes in microbiome composition to host health.^39^ For instance, distinct microbiome compositions have been linked to conditions such as inflammatory bowel disease (IBD), metabolic and brain disorders and aging.^40^ Novel intervention will likely need to consider varying environmental influences such as providing tailored approaches for specific population groups (*´mass personlisation´*) – an approach that is more practical than fully individualized solutions which have largely struggled to succeed in the market.

## 3. Microbial micronutrient sharing as a key characteristic of microbial ecosystems

**Figure 2.**
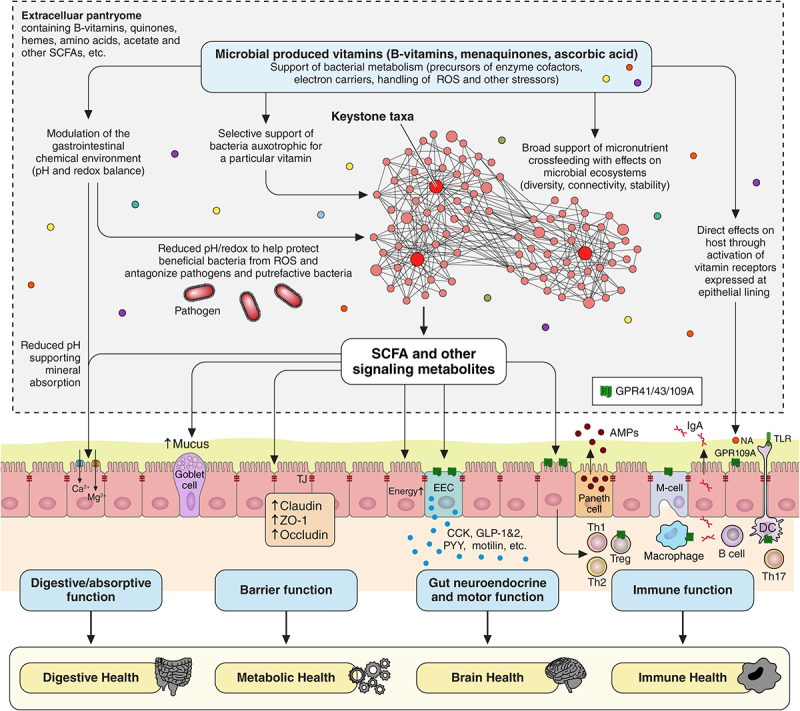
Overview of the hypothetical mechanism of action of colon-delivered vitamins. Colon-delivered vitamins may 1) exert broad effects on microbial ecosystems, such as changes in diversity, network performance, stability and connectivity, driven by their role as precursors of enzymatic cofactors (e.g., FAD, NAD) in bacterial cell metabolism; 2) provide selective support to bacteria that are auxotrophic for specific B-vitamins; 3) modify the gastrointestinal chemical environment by affecting pH and redox potential, thereby, directly protecting beneficial bacteria as well as influencing the balance between aerotolerant and strictly anaerobic species; 4) directly impact host health through interactions with intestinal lining receptors (e.g., niacin activating GPR109). These and other potential mechanisms may also interact with the fermentation of traditional carbohydrate-based prebiotics, potentially leading to more consistent host health benefits.

The classical model of cross-feeding views microbial interactions as sequential metabolic activities of various microorganisms exchanging lactate, acetate, or other products derived from macromolecule degradation.^41^ However, in addition to the classical carbon cross-feeding, recent findings indicate a significant role of cross-feeding of micronutrients essential for basic cellular metabolism, a finding which has hitherto largely been underappreciated.^42–44^ It appears that microorganisms are organized in highly interdependent networks constantly trading B-vitamins, quinones, amino acids, hemes, and other nutrients and bioenergetic molecules to promote growth and metabolism, to detoxify inhibitory molecules or to reduce reactive oxygen species (ROS) (Figure 2). A recent paper by Daisly et al.^42^ comprehensively summarizes the concept of nutrient sharing as the “Pantryome model of cross-feeding”. According to their model, *“distinct microbiomes develop interconnected and interdependent electron transport chains that rely on cooperative production and sharing of a bioenergetic machinery in the extracellular space”*. These communal nutrient resources represent an important subset of the microbial metabolome and are extensively shared between microbes thereby stabilizing bacterial communities but also making them more energy-efficient. They further argue that “*to form the leanest manufacturing networks possible, it is apparent that microorganisms must extensively share their resources in a communal pool within the extracellular space in order to optimize overall biosynthesis costs at the community level*”.^42^ Central to their model is the sharing of respiratory electron acceptors found outside the cell to enable anaerobic respiration via extracellular electron transfer. This complements the classical fermentation process which, in the absence of oxygen, was long believed to be the sole mechanisms for microorganism to generate ATP upon carbon breakdown. More recent studies have meanwhile built on this and identified a range of dietary- and host-derived metabolites used by diverse gut bacteria for anaerobic respiration.^45^

While prototrophic microorganisms can synthesize all of the essential micronutrients required for their growth and metabolism, auxotrophic microorganisms depend on external nutrient supply from the environment.^46^ Given that cooperative nutrient production is energetically more favorable than individualistic production, it is not surprising that in nature microorganisms are organized in highly interdependent ecosystems with most taxa being auxotrophic.^31,43^ Sharma et al.^44^ found through studies involving humanized gnotobiotic mice and anaerobic fecal cultures that the relative abundance of auxotrophic species in gut communities remained largely unchanged despite variations in diet or media lacking or greatly exceeding B-vitamins (approximately 30-fold above normal). This observation underscores the significant role of metabolic cooperation, specifically the exchange of B-vitamins, in maintaining the stability of gut bacterial ecosystems. It also suggests that nutrient requirements are likely hardwired into the microbial genome and therefore remain constant which contrasts with the dynamic nature of metabolic fermentative interactions among community members (i.e. substrate utilization patterns along the classical model of cross feeding) which can change over time based on diet and subtract availability.^47–49^ The existence of defined groups of potential partners with certain keystone taxa (as discussed later) may, therefore, have a unique and crucial role in microbial communities to allocate the necessary nutrients. Novel approaches to beneficially modulate these pathways are likely to have profound implications for microbial interactions independent of background diets or other confounding factors.

### 3.1. The role of B-vitamins as part of the ´pantryome´

B-vitamins primarily serve as precursors to a variety of highly conserved enzyme cofactors essential to the tricarboxylic acid (TCA) cycle, fatty acid oxidization, and other metabolic pathways.^50,51^ The long-standing consensus has been that B-vitamins auxotrophic species obtain the missing nutrient either by competing for these vitamins from the host’s diet or by acquiring them from host tissue leakage. Indeed, recent research investigating the link between dietary intake of B-vitamins and the composition and structure of the gut microbiome, based on the analysis of 97 colonic biopsies from 35 men, revealed significant differences in bacterial richness and composition.^52^ Lower consumption of folate and other B-vitamins was associated with reduced abundance of *Akkermansia, Roseburia*, and *Faecalibacterium* compared to higher consumption levels. However, what requires consideration is that under physiological conditions vitamins are absorbed efficiently in the proximal small intestine and do not reach the distal GI tract unless overdosed or poorly absorbed.^53^ A remarkable finding was made by Thiele and colleagues^54^ who systematically assessed the genomes of 256 common human gut bacteria for B-vitamins biosynthesis pathways. They discovered that gut bacteria produce and actively exchange B-vitamins among each other, thereby complementing and enabling the survival of organisms that do not synthesize B-vitamins in a ecosystem community approach. For example, only half of the genomes in the *Bacteroidetes* and *Firmicutes* phylum were predicted to be vitamin B12 (cobalamin) producers while all 14 *Fusobacteria* were predicted to synthesize cobalamin. Furthermore, all *Lactobacillales* were estimated to be non-producers, except six *Lactobacillus reuteri* strains that were previously known to have this pathway.^54^ Cobalamin is involved in pathways leading to SCFA production, as a cofactor for methylmalonyl-CoA mutase to produce propionate, and in one carbon metabolism leading to the production of acetate^55–57^ Vitamin B3 (niacin) biosynthesis pathways were found in a total of 63% of all the investigated microbial genomes.^54^ Niacin is also central to bacterial metabolism, as a classic NAD+ precursor, it serves primarily as electron/hydrogen acceptors in redox reactions mediating ATP generation, detoxification, and DNA repair.^58^ Vitamin B5 (panthothenic acid) is involved in various metabolic pathways such as the citric acid cycle, cell growth and fatty acid oxidation^59^ and was found to be present in nearly all investigated bacterial genomes.^54^

Of particular importance appears vitamin B2 (riboflavin). The biosynthesis pathway appeared to be highly conserved, yet it was absent in the majority of *Actinobacteria* genomes and half of the *Firmicutes* genomes. Notably, all non-producing organisms from these two phyla possessed the RibU riboflavin transporter gene suggesting their reliance on riboflavin-derived cofactors.^54^ Riboflavin, is a precursor of flavin mononucleotides (FMN) and flavin adenine dinucleotides (FAD) and both are co-enzymes in numerous oxidation and reduction reactions but also required by glutathione reductase (GSR), which protects cells from the harmful effect of ROS. As such, riboflavin has a key role not only as cofactors in energy metabolism but also as indirect antioxidant.^60^ In line with this, Harmsen and colleagues discovered that the gut anaerobe *Faecalibacterium prausnitzii* uses an extracellular electron shuttle of flavins and thiols to grow in oxygenated environments. They performed *in vitro* studies mimicking the gut mucosa with its steep oxygen gradient from the epithelium (where oxygen is delivered by the blood circulation) to the anaerobic lumen (where oxygen is consumed by microbial respiration). They found that *F. prausnitzii* can survive in moderately oxygenized environments by exploiting extracellular riboflavin to transfer electrons to oxygen to lower the redox potential.^61,62^ This provides a plausible mechanistic explanation of how certain anaerobes can survive in oxygenated environments where appropriate redox mediators are available.

### 3.2. Microbial micronutrient sharing and gut redox balance

Strict anaerobe commensals from the phyla *Bacteroidetes* and *Firmicutes* constitute the vast majority of microbes in the large intestine. They do not survive oxygen exposure but instead largely rely on anaerobic fermentation of complex polysaccharides for growth.^63^ Their strict sensitivity to oxygen explains why (despite being by far the most abundant phyla in the gut microbiome) they have paradoxically not been used for interventions so far on a large scale. ROS rapidly oxidize their sensitive enzyme systems as these microorganisms usually have no direct enzymatic means to protect themselves.^64–66^ In contrast, facultative anaerobic bacteria, which constitute only about 0.1% of the microbiota can tolerate small amounts of oxygen.^64,67,68^ This group includes *Enterobacteriaceae*, which, along with many harmless symbionts, encompasses familiar pathogens like *Salmonella, Escherichia coli, Klebsiella*, and *Shigella*. Interestingly, these bacteria have developed aerobic respiration mechanisms to function in the presence of oxygen and can often detoxify ROS effectively, giving them a competitive edge over beneficial commensals.^64,67,68^ Indeed 8 out of the 12 listed WHO priority pathogens are facultative anaerobes, demonstrating oxygen sensitivity to be a key determining factor in pathogen overgrowth in the gut microbiome.^64^

Microbial micronutrient sharing appears to be a crucial mechanism for strict anaerobe commensals to cope with redox stress, thereby stabilizing fluctuations in redox balance. However, although disruption of anaerobicity and related oxidative stress represents a hallmark of various diseases,^69,70^ it’s important to understand that maintaining redox balance goes beyond merely neutralizing harmful-free radicals. Rather, it involves a complex set of processes involved in the maintenance of both the electrophilic and nucleophilic tone and their balance across the dynamic environmental niches. In fact, emerging evidence suggests that ROS are vital physiological signaling molecules with a crucial impact on the proper functioning of diverse signal transduction pathways. As such, redox signaling plays a critical role in the maintenance of gut homeostasis with significant effects on the normal functioning of the gut microbial ecosystem as well as various gut-related cell types, gut barrier integrity, and the gut immune system.^71–74^

Under healthy conditions, the gut microbiome actively participates in the regulation of the gut redox state. For example, it has been speculated that commensal bacteria can induce the production of ROS by intestinal epithelial cells reflecting an important mechanism in the defense against pathogens, to support mucosal healing or modulate innate immune signaling.^73^ Additionally, the gut microbiome indirectly influences oxygen balance through the secretion of SCFAs, which are metabolized by colonocytes in an oxygen-consuming process.^75–77^ In contrast, under pathophysiological conditions, oxygen and redox balance can shift so that either cellular signaling is impaired and/or oxidative damage is promoted leading to detrimental effects on mucosal integrity and epithelial barrier function. This is referred to as ´oxidative stress´ and is often observed with pathogen infections, antibiotic treatment and inflammatory conditions of the large bowel such as Crohn´s disease (CD) or ulcerative colitis.^78–81^It is usually accompanied by microbiome dysbiosis which is characterized by a marked decrease in strict anaerobic bacteria and an increase in facultative anaerobic bacteria.^82–84^An interesting hypothesis was suggested by million and Raoult et al. ^85^ who showed that fecal redox potential is related to the Metagenomic Aerotolerant Predominance Index (MAPI), defined as the natural logarithm of the ratio of the relative abundances of aerotolerant to strict anaerobic species. While the MAPI was found to be negative in healthy individuals (anaerobic predominance), it was positive in children with malnutrition (aerotolerant predominance). Moreover, and despite high variability in redox potential measurements, the MAPI was significantly higher in individuals with positive fecal redox potential than in individuals with negative fecal redox potential.^85^ A similar finding was recently made by Kort et al.^86^ who reported higher levels of oxygen tolerant species and a trend for an increased MAPI index in a language impaired vs. language non-impaired group of three-year-old rural Ugandan children. Thus, protecting strict anaerobic commensals from oxidative stress via the supply of microbial micronutrients such as riboflavin (which is critical for GSR functioning as mentioned above), or antioxidant acorbic acid which is also produced by gut bacteria ^87,88^ may be a promising strategy to effectively help stabilizing fluctuations in redox balance and gut microbiome homeostasis (Figure 2).

This appears particularly important given the recent findings of a decrease in microbial vitamin production in oxidative stress-related conditions including IBD, malnutrition, and type 2 diabetes mellitus.^89–91^ In addition, Belda et al.^92^ found that severe obesity was associated with an absolute deficiency in bacterial biotin producers and transporters whose abundances correlated with host metabolic and inflammatory phenotypes. Further, Wang et al. systematically investigated the relationship between gut microbial vitamin production and factors related to diabetes and cardiometabolic health in a deeply phenotyped cohort (Lifelines-DEEP) including 1135 subjects. They found that blood glucose-related factors, lipids, circulating inflammation, and fecal SCFA were associated with gut microbial vitamin production and revealed a mediatory role for microbial vitamin B1/B2 production on the influence of fruit intake on diabetes risk.^93^ Finally, a recent study in centenarians in Jiaoling, China suggested that an age-related gut-resident *Lactobacillus* and its metabolite antioxidant L-ascorbic acid contributes to health and longevity ^88^

In summary, another approach to beneficially modulate microbial networks involves leveraging naturally evolved metabolites including vitamins produced by commensal microorganisms. Similar to how polyphenols protect plants from insect attacks or plant cryoprotectants shield against cold, gut microbes may have evolved to produce and share B-vitamins and other metabolites to protect themselves from stress and promote growth and survival. These metabolites either *´condition´* the gut environment (e.g., lactate production lowers pH, thus, favoring the survival and growth of *lactobacilli* and other beneficial bacteria) or directly neutralize harmful agents (e.g. riboflavin acts as an electron shuttle to protect against oxidative stress). Conversely, reduced microbial vitamin production may indicate a decreased natural resilience of commensal anaerobes to oxidative stress. We hypothesize that supporting microbial micronutrient sharing has the potential to benefit microbiome and host health across variable microbiome profiles, given the pathways are genetically conserved, and, that their influence extends also by modulating the GI chemical colonic environment (Figure 2).

## 4. Keystone taxa as another key characteristic of microbial ecosystems

The alimentary tract is not a homogenous ecosystem but comprises multiple environments, each corresponding to different sections from the mouth to the rectum and anus. Each ecosystem is characterized by specific environmental parameters such as transit time, pH, bile, and oxygen levels.^94^ The colon, which harbors the largest number of microorganisms as discussed above, is a predominantly anaerobic environment with a pH ranging from 5.5 to 7.0 and slow transit time of up to 46 h (for the proximal part) and 80 h (for the distal colon) although this can vary substantially between and within individuals.^95^ Accumulating evidence suggests that particularly gut transit time is a key factor in shaping gut microbiota composition and activity with prolonged gut transit time linked to higher fecal pH, lower fecal water content, increased microbial cell density and diversity, and a shift in microbial metabolism from saccharolysis to proteolysis.^95^

Growing evidence indicates that microbial communities along different niches of the GI tract contain keystone taxa, which drive local community composition and function regardless of their abundance, a phenomenon similar to other large-scale ecosystems.^39,96–99^ The term ‘keystone species’ was introduced first in 1966 by American ecologist Robert Paine, who identified a particular sea star as a crucial predator regulating biodiversity in the tidal plains of Washington State. This sea star kept mussel and barnacle populations in check, thereby supporting healthy populations of seaweeds and the communities that fed on them, such as sea urchins and sea snails. Similarly, gut microbial keystone taxa have been defined as “*highly connected taxa that individually or in a guild exert a considerable influence on microbiome structure and functioning irrespective of their abundance across space and time”*. They are suggested to exert a crucial role in microbial communities, and their removal to cause a dramatic shift in microbiome structure and functioning.”^96^

It is likely that the next generation of microbiome-based therapies will be about the identification and selective modulation of these keystone species and their interactions with other important species in microbial ecosystems. Especially, when reestablishing gut microbial balance, keystone species may play an essential role.^94^ This is illustrated in recent studies identifying bacterial taxa involved in post-antibiotic recovery in human cohorts. Chng et al. ^99,100^ found that a succession of primary colonizers prepares the environment for later dominant species, which thrive on the breakdown products of the pioneer species. The study identified seven bacterial species as primary colonizers with the metabolic ability to extract carbon and energy from mucin and complex dietary carbohydrates, thus, occupying the base of the food chain. Although most of the identified primary colonizers were abundant species, primary colonizers are not necessarily the most abundant species, because there is no correlation between abundance and physiological importance. Rather, some low abundance species may carry essential functions that support the growth of other dominant species.

A list of prominent keystone taxa of the colonic microbiota has been described recently based on the identification of critical enzymes involved in cross-feeding interactions including *Akkermansia muciniphilia*, *Bifidobacterium longum* or *Faecalibacterium prausnitzii*.^39,94,97,98^ Each carries a specific function (mucin degrader, degradation of complex carbohydrates such as HMOs, butyrate production, etc) that is essential for microbiome balance and whose disruption has been associated with disease development. Keystone taxa are likely to play also a crucial role in maintaining ecosystem balance through the production of essential vitamins as disucssed above, however, future research is needed to better understand the precise role of microbial vitamin sharing and interdependencies of prototrophic vs. auxotrophic bacteria.

What requires consideration is that today, there is still a lack of an effective framework for identifying keystone taxa from the available high-throughput sequencing without the challenging task of reconstructing the detailed network of other ecological principles that influence the overall dynamics of microbial communities.^101^ This includes the complex interaction among various microbial species influenced by factors such as biogeography of the gut. As mentioned above, while the large intestine contains the highest number of microbes, the small intestine hosts only a limited range of microbes including *Gemella, Streptococcus, Escherichia and Veillonella spp*^102^ based on the challenging environmental conditions. There is also distinct luminal and mucosal microbial communities, each characterized by different important functional species. The mucosal-associated colonic microbiome is particularly enriched with members of *Akkermansia*, *Bacteroidaceae* and *Lachnospiraceae* some of which are specialized in utilizing mucin.^103,104^ Similar to the lumen, the mucosal microbiome exhibits significant variation along the gut, with less than 60% of species shared across its length.^105^

Microbiome maturation is another factor influencing ecosystem dynamics. At birth, vaginal delivery facilitates the vertical transmission of microbes like *Bacteroides* and *Bifidobacterium* from the maternal gut, shaping the infant’s initial ecosystem.^106^ In contrast, cesarean delivery disrupts microbial transfer which often delays commensal acquisition and increases exposure to hospital-acquired, potentially multi-drug-resistant bacteria.^107^ As discussed above, *Bifidobacterium* metabolize complex HMOs that infants cannot digest, unlike formula-fed infants who typically exhibit higher microbial diversity with elevated levels of potentially pathogenic species like *Clostridioides difficile* and *Escherichia coli* ^19,108^ As solid foods are introduced, genera such as *Bacteroides*, *Veillonella*, *Roseburia*, and members of the *Lachnospiraceae* family gradually begin to establish.^109^ The large variability of microbial communies in adults has led to the proposal of functional “enterotypes” based on dominant species from *Ruminococcus, Bacteroides*, or *Prevotella*.^110^ However, the existence of true microbial clusters remains debated and whether there is enterotype-specific keystone species or whether relative importance of keystone species vary with gut biogeography or microbiome maturation is currently unkown and requires further investigations.

Besides the relative importance of specific (keystone) taxa, also overall microbiome diversity is widely recognized as a key characteristic of microbial ecosystems. Although this is sometimes criticized for being overly simplistic (since it is not diversity itself, but rather the metabolic functions that benefits the host), the overall positive correlations between microbiome diversity and host health suggest that a more diverse community can occupy a broader range of functional niches with better utilization of by-products of one another’s metabolic processes.^111^ These interactions may extend beyond keystone species with benefits arising from the communities collective metabolic activities with a large and dense metabolic network making pathogen colonization increasingly difficult (a phenomenon known as “niche exclusion”).^112^

Taken together, keystone taxa may play a critical role in maintaining the structure and function of microbial communities making them valuable targets for innovative biotic interventions. However, there are various layers of complexity that yet complicate the understanding of their relative importance across life stages, diets, and environmental exposures suggesting that a fluid interplay of microbial species adapting to shifts in host and external conditions are crucial for maintaining host health.

## 5. The importance of the gut barrier

The intestinal barrier plays a fundamental role in host health and disease and is tightly linked to gut microbial health. It is composed of several elements to form a physical and immunological defense barrier including 1) the outer mucus layer with the commensal gut microbiota, antimicrobial proteins and secretory immunoglobulin A; 2) a central cell layer with epithelial cells, and 3) the inner lamina propria where innate and adaptive immune cells reside such as T cells, B cells, macrophages, and dendritic cells.^113^ In humans, the intestinal barrier represents the largest interface of the body with the environment, with about 400 m^2^ constantly exposed to nutrients, drugs, toxins as well as microorganisms resulting in a substantial inflammatory and pro-oxidative potential.^72^ Maintenance of a functional gut barrier is, therefore, crucial for overall host health in order to prevent systemic inflammation and oxidative stress.

SCFA are key molecules linking barrier integrity to gut microbial health and redox balance. They are produced by commensal bacteria via fermentation of polysaccharides with acetate and lactate being the main metabolic end products of mostly bifido- and lactic acid bacteria. Acetate and lactate are utilized further by other microorganisms to produce propionate and butyrate.^114^ Butyrate is the most important regulator of tight junction proteins (e.g. claudin-1 and zonula occludens-1 (ZO-1), thereby directly promoting epithelial barrier function.^115^ It is recognized by intestinal epithelial cells via the G-protein coupled receptor GPR41/43/109A, however, GPR´s link SCFA to many other intestinal and extraintestinal functions such as the secretion of peptide hormones including glucagon-like peptide 1 or peptide YY both being involved in appetite and blood glucose control. Butyrate also stimulates MUC2 gene expression, which results in an increase in mucus production by goblet cells. Finally, butyrate serves as important energy sources of ATP for colonocyte function and is metabolized in an oxygen consuming reaction, thus, contributing directly to a low-oxygen environment.^75–77^

The link between fiber consumption, SCFA production and gut redox balance was recently investigated by Belenky and coworkers.^116^ They found that dietary fiber supplementation (a cocktail of 7 plant fibers including cellulose, levan, dextrin, pectin, inulin, beta-glucan, arabinoxylan) *vs*. glucose protected mice from antibiotic-induced dysbiosis by modulating gut redox potential. Their experiments were based on the hypothesis that with western diets low in fiber, there is limited carbon sources available for commensal microbes, therefore, they metabolize host-derived carbon from mucosal linings in the intestine. This increases the potential for gut inflammation and loss of barrier integrity. Furthermore, it changes microbiome composition by selecting for bacteria that thrive in inflammatory and more aerobic environments favoring metabolic reactions with higher redox potential. In contrast, fiber-rich diets select for microbes that metabolize complex polysaccharides using fermentative metabolism. This results in an increase in SCFA production which are metabolized by colonocytes in an oxygen consuming reaction resulting in a more anaerobic environment with lower redox potential that favors a more healthy microbial ecosystem.

## 6. Novel solutions targeting health from the gut based on microbial micronutrients and precision prebiotics

### 6.1. Microbial micronutrients

Despite the fact that particularly pre- and probiotics are clinically well established for a variety of health benefits, they often suffer from high interindividual variability in clinical outcomes due to differences in host physiology, background diets, individual host microbiome compositions, and other environmental influences. The supply of microbial micronutrients (alone or combined with pre and probiotics) to support and balance microbial ecosystems may offer a more robust and reliable approach across different subjects and environmental backgrounds.

In fact, the 2017 ISAPP prebiotic consensus definition already included vitamins as “substances” that, although not traditionally defined as ‘prebiotic’, can affect the composition of the microbiota.^11^ A follow-up paper from ISAPP´s 2019 Annual Meeting on the future of probiotics and prebiotics also referred to vitamins as organic molecules that are essential micronutrients to support an organism’s metabolism.^13^ As such they were considered *´overlapping and adjacent to the current boundaries of prebiotics´* with an emerging role to impart beneficial health effects upon the host. The paper concluded that more research is warranted to better understand the metabolic pathways of vitamin utilization in the colonic microbiome.

In 2020, Soto-Martin et al.^117^ confirmed that many of the dominant commensal butyrate producers such as *Ruminococcaceae, F. prausnitzii* and *Subdoligranulum variabile* are auxotrophic for most of the B-vitamins in a combination of *in silico* analyses of vitamin biosynthetic pathways and *in vitro* growth tests. Emerging evidence arrives also from human clinical studies indicating that vitamins can effectively modulate the gut microbiota. For example, in a pilot open-label study in healthy humans, oral supplementation of 100 mg riboflavin for 14 days increased the number of strict anaerobe *F. prausnitzii*, while the number dropped again (although not to baseline levels) after a 1-week washout period. In addition to this increase, there was an increase in anaerobe *Roseburia* species, and a decrease in *E. coli* suggesting an improvement in the anaerobic conditions and gut redox balance.^118^ The exact amount of dietary riboflavin that reached the colon with a 100 mg oral dose remained, however, unknown. Absorption takes place predominantly in the proximal small intestine through an active, carrier-mediated, saturable transport process that is reported to be linear up to 30 mg riboflavin in a meal.^119,120^ Therefore, some riboflavin likely reached the colon; although colon-targeted delivery systems would provide a more efficient method for reliably delivering vitamins to the large intestine.^53^ In a follow-up, clinical study in 70 patients with CD with varying disease activities, oral riboflavin (100 mg/day) for 3 weeks significantly decreased serum levels of inflammatory markers associated with a decrease in *Enterobacteriaceae* in patients with low fecal calprotectin as determined by fluorescence in situ hybridization (FISH). ^121^ Yet another randomized, placebo-controlled trial in healthy subjects investigating the effect of 2 weeks of either 50 mg or 100 mg/d of oral riboflavin supplementation failed to show an effect on *F. prausnitzii*. However, there was an increase in the complexity and stability of bacterial networks as well as fecal acetate and butyrate concentrations.^122^

Besides overdosing, effects on microbiome composition and metabolic activity were investigated also following colon-targeted delivery of vitamins. Pham et al.^123^ found in healthy subjects that colon-delivered riboflavin and ascorbic acid over 4 weeks increased microbial alpha diversity, relative abundance of *Coprococcus* and *Alistipes* as well as fecal SCFA concentrations. When nicotinic acid was delivered to the colon in another clinical study, it produced a significant increase in the abundance of *Bacteroidetes*, which in the absence of systemic side effects was associated with an improvement of biomarkers for systemic insulin sensitivity and metabolic inflammation in healthy subjects.^124^ Further large-scale clinical studies involving colon-delivered vitamin B2, vitamin C, and combinations of B2, B3, and C are currently underway in healthy elderly individuals and those with IBD. These studies will provide deeper insights into the effects of colon-delivered vitamins on microbiome health and long-term safety (ClinicalTrials.gov numbers NCT05803811, NCT05598619 and NCT04913467).

None of the reported studies have so far indicated any adverse event which seems not surprising given that even under physiological condition, vitamins may reach the large intestine. B-vitamins are naturally present in the food matrix with B1 to B6 contents found in nearly 800 common food items and B9 to B12 in nearly 300 foods.^125^ Plant-based foods including legumes, vegetables, nuts, seeds, fruits, and cereals are the primary source and also include dietary fiber that can bind vitamins and phytochemicals to influence bioaccessibility and bioavailability facilitating their natural delivery to the colon.^125–128^ In addition to food, there is microbial vitamin production with approximately 12.5% of gut bacteria capable of producing all eight B-vitamins leading to a constant exposure of the colon to these nutrients.^54^ Finally, commercially available vitamin products have been widely used for decades, often exceeding intestinal absorption limits, which enables B-vitamins to reach distal parts of the intestine without any reported safety issues. One could specuate that small amounts spilling over into the ileum and colon may even play an important role in their recognized health benefits as well as help to clear the intestine from pathogens.^129^ However, this spillover can vary greatly among individual depending on factors such as differences in bowel length, the influence of fasted versus fed states, variations in food matrices, and other physiological variables suggesting the need for microbiome targeted technologies.^130,131^

There is also research in experimental rodents showing that B-vitamin supplementation supports gut microbiome homeostasis.^132^ In a colitis mouse model, riboflavin ameliorated intestinal inflammation, which was linked to improved intestinal barrier function (by increasing expression of tight junction proteins) and an increase in SCFA and the relative abundance of *Actinobacteriota, Desulfobacterota* and *Verrucomicrobiota*.^132^ Moreover, when high-fat diet-fed mice were supplemented with FOS and biotin, Belda et al.^92^ observed improvements in microbiome diversity and an increase in bacterial production of biotin and other B-vitamins while limiting weight gain and glycemic deterioration. Taken together, these findings underscore the potential of targeted vitamin interventions to support and foster microbial micronutrient sharing in a colon niche-specific manner with effects on host health.

The full potential of colon-delivered vitamins to improve microbiome and host health remains, however, constrained by a number of factors. This includes the lack of understanding of the precise mechanisms for modulating the gut microbiome, uncertainties around optimal dosages and delivery formats, and limited documentation of associated health benefits. To address the issue of delivery formats, we recently validated a double-layer coated multi-unit particle system (MUPS) with a diameter of approximately 730 microns, composed entirely of food-grade materials.^133^ The formulation includes shellac as the outer layer, alginate as the inner layer, cellulose as the core, and riboflavin as the active ingredient. The MUPS were evaluated for colonic delivery in three well-established *in vitro* digestion and fermentation models: USP Apparatus 3 and the TNO Intestinal Models 1 and 2 (TIM-1 and TIM-2). All models confirmed the structural integrity of the MUPS under simulated gastrointestinal conditions, with approximately 90% of the active ingredient released under simulated ileal-colonic conditions. Additionally, the TIM-2 model demonstrated that riboflavin-loaded MUPS positively influenced microbiome composition by enhancing the production of acetate and butyrate. Following the recent announcement of the European Commission to restrict intentionally added microplastics to products,^134^ the materials used in the described formulation, therefore offer an environmentally friendly alternative to often applied methyl acrylate-based coatings.

Regarding optimal dosages, two ongoing clinical studies involving vitamins B2 and C are specifically designed to assess dose-response effects and will provide valuable insights (ClinicalTrials.gov numbers NCT05803811, NCT05598619). In contrast, the demonstration of microbiome-related host health benefits and the underlying mechanisms of action are likely to remain an area for ongoing exploration. This is a challenge shared also by other biotics given that pinpointing the exact mechanisms of action is complex as it likely involves a range of different effects as summarized in Figure 2. First, colon-delivered vitamins may exert broad effects on microbial ecosystems, such as changes in diversity, network performance, and connectivity, driven by their role as precursors of enzymatic cofactors (e.g., FAD, NAD) in bacterial cell metabolism.^46^ Second, they may modulate the gastrointestinal chemical environment by influencing pH and redox potential with effects on the ratio of aerotolerant *vs*. strict anaerobic species as outlined above. Besides vitamin C’s antioxidant properties that are well-documented,^135^ riboflavin is crucial for the functioning of the GSR which protects cells from oxidative damage caused by ROS.^60^ Third, certain bacteria, which are auxotrophic for a particular B-vitamin may benefit directly from the provision of these micronutrients, selectively enhancing their growth and performance.^31,43,54^ Forth, colon-delivered vitamins may have direct effects on host health through activating vitamin receptors expressed on the epithelial lining. For example, niacin activates the GPR109 receptor resulting in anti-inflammatory effects.^136,137^ In contrast, loss of GPR109A is associated with reduced levels of tight junction proteins and increased intestinal permeability.^138^ Interestingly, this receptor is also activated by butyrate, indicating potential beneficial overlapping mechanisms.^139^ Lastly, there might be interaction effects with classical carbohydrate-based prebiotics, and it will be intriguing to study whether colon-delivered vitamins can influence classical fermentation dynamics, potentially creating synergistic effects (Figure 2). Further research into these mechanisms will help establish evidence for host health benefits, such as improving conditions associated with microbiome dysbiosis, as seen after antibiotic or other medication use, aging, stress, or metabolic diseases.

### 6.2. Precision prebiotics

Besides supporting microbial micronutrient sharing, metabolic support through carbohydrate-based precision prebiotics is another avenue to beneficially modulate the gut microbiome in a more targeted manner. This aligns well with the classical “prebiotic concept” defined by ISAPP as *“substrates that are selectively utilized by host microorganisms conferring a health benefit”*.^11^ However, while earlier interpretations of ‘selectively’ mainly referred to *Lactobacilli* and *Bifidobacteria*, it is now recognized that beneficial effects extend beyond these two taxa including many of the earlier mentioned keystone species.^96,99,140^ Consequently, there is a growing list of novel or “candidate” prebiotics that are discussed including xylooligosaccharides (XOS), resistant starch, polydextrose or HMOs with demonstrated prebiotic potential *in vitro* ^141^. Although their host health benefits are sometimes less well established compared to the classical prebiotics such as FOS, inulin and GOS, they represent a promising evolution of the prebiotic concept. To support this, a panel of experts recently built upon the key concepts outlined in the 2017 prebiotic consensus statement, offering additional guiding principles for the development of novel prebiotics in order to improve scientific substantiation, product labeling, and consumer communications.^142^

Interestingly, recent research shows that many classical prebiotics are in fact less selective than originally thought, as numerous bacterial taxa can access and degrade them.^143–145^ For example, in mice, FOS feeding decreased *Firmicutes* and increased *Bacteroidetes*, but also changed 102 distinct taxa, 16 of which displayed a 10-fold change in abundance.^144^ Likewise, in a study in healthy adults, GOS was reported to specifically increase *Bifidobacterium spp*. although only 11 of 18 subjects showed actual increases in *Bifidobacterium spp*.^146^ In another study of 43 individuals supplemented with resistant starch, only 22 individuals responded with a butyrate increase, while 21 individuals responded with a reduction or no changes in fecal butyrate concentration.^147^ These findings may explain the high inter-individual variability in host health benefits observed with these classical prebiotics but also point to the importance of measuring the effect of prebiotics on microbiome function rather than composition only.

**Figure 3.**
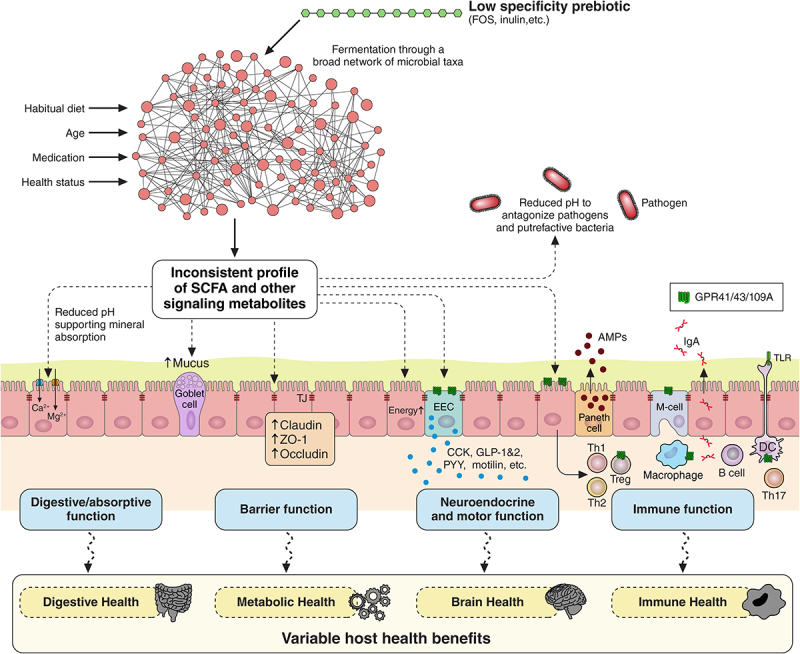
Schematic of the hypothetical mechanisms of action of low specificity prebiotics on gut microbiota composition and metabolic activity and related host health benefits. Low-specificity prebiotics such as frucotoligossachirides (FOS) and inulin are easily accessible and can be utilized by a broad range of microbes. As a result, their fermentation dynamics are largely influenced by the composition of an individual’s gut microbiota which varies based on habitual diet, age, medication, and health status. This variability can result in an inconsistent production of short-chain fatty acids (SCFAs) and other metabolites with variable host health benefits.

**Figure 4.**
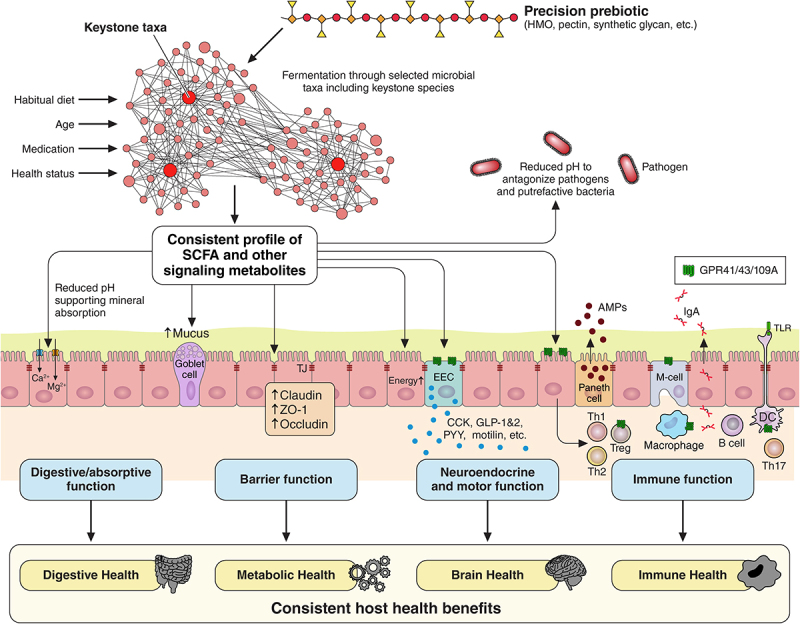
Schematic of the hypothetical mechanisms of action of precision prebiotics on gut microbiota composition and metabolic activity and related host health benefits. Precision prebiotics and high-specificity fibers (i.e., fibers with complex chemical structures and a variety of sugars and linkage types) are likely accessible to only a limited number of bacteria including keystone species. This selectivity may lead to a more predictable and consistent production of short-chain fatty acids (SCFAs) and other metabolites with reliable host health benefits.

An intriguing hypothesis on dietary fiber selection for predictable shifts in gut microbiota was proposed recently by Cantu-Jungles & Hamaker.^143^ They argued that with low-specificity fermentable fibers such as FOS and inulin, fermentation responses largely rely on the gut microbes’ ability to compete among each other to utilize these fibrers. In other words, because low-specificity fibers are easily accessible and can be utilized by many colonic microbes, the resulting competitive pressures lead to a response that varies based on an individual’s gut microbiota community structure influenced primarily by environmental factors such as habitual diets, age, medications, and health status (Figure 3). Conversely, high-specificity fibers (i.e., fibers with complex chemical structures and a variety of sugars and linkage types) may be accessible to only a few bacteria, reducing competition and hypothetically leading to a more predictable and consistent prebiotic response across populations^143^ (Figure 4).

Examples of such high-specificity prebiotics may include beta glucans. A fungal insoluble -(1–3)-linked beta-glucan appeared highly specific for *Anaerostipes* in an *in vitro* human fecal fermentation, increasing their abundance from 0.5% to approximately 24% within 24 h, with few other gut microbes able to compete and utilize this glycan.^148^ Also HMOs may be regarded as high-specificity prebiotics based on their selective effect on *Bifidobacteria*, particularly in early life to support early founder strains of the infant microbiome as mentioned earlier.^18,19,149,150^ Using the novel ex vivo SIFR® technology, Bajic et al.^151^ found that 2‘FL/LNnT selectively increased *Bifidobacteria* in both, 6-year-old children and adults, while this was not the case with inulin and FOS that enhanced *Bifidobacteria* only in adults. Using the same system, also a carrotpectin derived polysaccharide, i.e. rhamnogalacturonan was recently found to be highly specific by lowering interpersonal compositional differences.^152^ This was due to the selective stimulation of taxa that were consistently present among human adults, including OTUs related to *Bacteroides dorei/vulgatus* and *Bifidobacterium longum*, both suspected keystone species as well as *Bifidobacterium adolescentis* and butyrate-producing taxa such as *Blautia sp*. or *F. prausnitzii*. In contrast, both inulin treatments increased interpersonal compositional differences. Interesting was also that these effects were observed already at low doses of only 0.3 g/d and although this was observed only under *ex vivo* conditions, it suggests that selective prebiotics may address one of the main challenges of traditional prebiotics that typically need to be consumed in high (>5 g) doses to exert health effects.

An exciting area of recent research also suggests synthetic glycans as precision prebiotics, which could be developed to recapitulate features of the chemical complexity of dietary fiber by controlling reaction conditions including types of monosaccharides, bond types, and degrees of polymerization. Tolenen et al.^153^ recently showed that fermentation of a range of synthetic glycans resulted in specific shifts in taxonomic and metabolite profiles, which were not observed with natural reference glycans commonly found in diets. Synthetic glycan fermentation specifically enriched abundances of *Lachnospiraceae*, *Parabacteroides*, and *Roseburia*, a key butyrate producer which is depleted in Crohn disease subjects^154^ as well as *Fusicatenibacter* which is depleted in active ulcerative colitis.^155^ Interestingly, synthetic glycans also conferred a selective growth benefit for commensals over gut pathogens potentially increasing their safety for at-risk populations.^153^

However, it is important to consider that the development of synthetic glycans still faces several limitations, including insufficient mechanistic evidence, a limited understanding of precise microbial responses, and unresolved regulatory and safety concerns, particularly regarding their long-term effects on microbiome and host health. For instance, more research is needed to determine whether superior selectivity in microbial responses benefits host health or negatively impacts microbial diversity, and what is the impact on overall microbial community dynamics in the long term. It also remains uncertain whether the high specificity can actually withstand individual variations in microbiome composition and environmental factors. In terms of product safety, comprehensive investigations are warranted to ensure synthetic glycans do not promote opportunistic pathogens, harm beneficial taxa, or interact negatively with host cell receptors. Safety assessments should account here for diverse populations including infants and other vulnerable groups and will need to be compliant with the regulatory frameworks including Novel Food Regulations in the EU and GRAS/self-GRAS in the U.S. Finally, the development of next-generation, synthetic glycans should also prioritize sustainable sourcing and manufacturing practices, aligning with the societal efforts to promote planetary health. As such, synthetic glycans could be synthesized from plant-based monosaccharides including glucose, xylose, or others with sustainability influenced by farming practices like reduced pesticide use and water conservation. While the production process may involve chemical synthesis which can impact the environment through energy usage and emissions, adopting energy-efficient and renewable energy-based manufacturing could help to mitigate this. Therefore, when compared to more resource-intensive traditional fibers, synthetic glycans could become a more sustainable choice, especially when sourced from organic materials and produced using circular systems that minimize waste. As mentioned earlier, Hutkins et al. ^142^ recently summarized several of these aspects, offering a guiding perspective for the development of novel prebiotics.

### 6.3. Synergism between microbial nutrients and prebiotics

Interestingly, there seems to be synergies between providing microbial micronutrients and prebiotics, both at microbiome and host level. While further empirical research is required to definitively confirm a causal link, early findings provide compelling indications that such a connection may exist: 1) A recent clinical study investigating the efficacy of β-fructans (FOS and inulin) in preventing relapse in adult IBD patients found that although there was a positive impact in many patients with IBD, β-fructans (15 g/d over 6 months) could not prevent symptomatic relapses in a subgroup of patients with UC in remission.^156^ Surprisingly, the fructan fibers remained intact in these patients who were lacking microbial fermentative activities for fructan digestion, with subsequent induction of pro-inflammatory responses. The authors also found that the fructan fibers induced immune responses correlated with the endogenous microbial ability to synthesize riboflavin so that decreased microbial riboflavin synthesis correlated with increases in pro-inflammatory responses to β-fructans. This observation was further validated through demonstrating that riboflavin was significantly lowered in stool samples collected from UC patients who relapsed upon β-fructan. Based on these data, the authors suggested a critical link between fiber-degrading microbes, the production of SCFAs, microbially produced riboflavin, and the inflammatory response to dietary fibers. 2) When high-fat diet-fed mice were supplemented with FOS and biotin, they showed improvements in microbiome diversity and an increase in bacterial production of biotin and other B-vitamins while limiting weight gain and glycemic deterioration.^92^ 3) A study in Chinese centenarians showed that an age-related gut-resident *Lactobacillus* and its metabolite L-ascorbic acid contributes to health and longevity^88^ 4) A recent study with high-fat diet treated mice found that dietary fiber (Dendrobium officinale polysaccharide) supplementation induced the growth of *Parabacteroides distasonis* and, therefore, protected against insulin resistance via microbial production of nicotinic acid that activates intestinal GPR109a to enhances intestinal barrier function.^157^ 5) A recent study conducted on gnotobiotic and germ-free mice fed diets with varying fiber content also yielded intriguing findings. Analysis of cecal contents showed that fiber deprivation consistently reduced the levels of microbiota-produced B-vitamins. This reduction was not due to decreased biosynthesis but rather an increased microbial utilization of specific B-vitamins under fiber-free conditions, as indicated by metatranscriptomic analyses. Additionally, fiber deprivation disrupted immune homeostasis while supplementing the diet with inulin restored both, the availability of microbially produced B-vitamins, and immune function.^158^ 6) As mentioned earlier, riboflavin appears to be particularly utilized by the commensal, butyrate-producing and inulin-fermenting microbe *F. prausnitzii*.^61^ The recent findings demonstrating the capacity of *F. prausnitzii* to degrade β-fructans, particularly inulin, with resulting anti-inflammatory cell viability-promoting effects^159^ suggests a potential role for riboflavin in determining microbial inulin degrading capacity of the keystone species *F. prausnitzii*.

Intriguinly, population studies have firmly established that diet-related factors, including the consumption of plant edibles, play a significant role in the variability of the human microbiome across individuals.^35,36^ Plant-based foods such as legumes, vegetables, nuts, seeds, fruits, and cereals are key sources of dierary fiber that can bind vitamins and phytochemicals suggesting that these foods could participate in preserving a healthy intestinal ecology by providing micronutrients to the colon. In other words, dietary fiber may influence bioaccessibility and bioavailability of phenolic compounds and vitamins facilitating their natural delivery to the colon.^125–128^ Therefore, the proposed importance of co-delivering microbial nutrients and indigestible glycans may align with the known health benefits of plant-rich diets high in dietary fiber.

## 7. Predictive bioinformatics to investigate microbial ecosystem dynamics

Most microbiome studies are empirical, using either case-control or randomized designs.^160,161^ Microbiome composition is typically analyzed through DNA sequencing, using one of two main methods: amplicon sequencing, often targeting the 16S rRNA gene, or whole genome sequencing (WGS). Amplicon sequencing is a cost-effective method, particularly useful in samples with host DNA contamination, but its limited taxonomic resolution and inability to assess functional potential often constrain its applications. Predictive tools like PICRUSt^162^ can infer functions from taxonomic data, but their scope is limited. In contrast, WGS offers higher taxonomic resolution and directly measures the functional potential of microbial communities. Recent advancements in bioinformatics have further expanded the capabilities of WGS by enabling the assembly and reconstruction of entire genome sequences, referred to as metagenome-assembled genomes (MAGs). MAGs offer substantial potential for predicting nutrient-related functional activities of microbial species, as they integrate taxonomic and functional analyses derived from WGS data.

Having taxonomically annotated genomes and information on the functions encoded (through gene annotation) allows for the prediction of which compounds a species can metabolize. This makes it possible to predict which species would increase or decrease in abundance given a particular metabolite. From WGS alone, it is possible to identify the presence or absence of specific functions, but not the species being modulated. This approach is similar to the one already discussed by Magnúsdóttir *et al.* ,^54^ which identified genome capabilities for B-vitamin biosynthesis using reference genomes, whereas MAGs offer the advantage of capturing greater microbial diversity. A critical step in this process is the accurate annotation of predicted gene sequences. While numerous tools are available, such as HMMER^163^ and DIAMOND,^164^ the quality of the database used plays a crucial role in achieving accurate annotations. A robust database requires not only a large set of annotated genes but also a high level of curation and quality. This remains a significant challenge in human microbiome research, as many genes still lack functional annotation. For instance, a large study analyzing the human gut microbiome revealed that 40% of the protein-coding sequences have no functional annotations.^165^

With regard to nutrient utilization, several specialized database resources provide valuable insights. The CAZy database focuses on enzymes involved in carbohydrate metabolism, while PULs (Polysaccharide Utilization Loci) highlight genomic regions for carbohydrate uptake and utilization. The EC (Enzyme Commission) classification links enzymes to their specific reactions, and KEGG KOs (Kyoto Encyclopedia of Genes and Genomes Orthology) integrate genes into functional pathways, enabling metabolic network interpretation. Together, these resources facilitate mapping microbial functions to nutrient cycling and dietary metabolite processing.

Of particular interest, in light of micronutrient sharing, is the use of the Transporter Classification Database (TCDB) to model the exchange of substances and identify nutrient-specific transporters (e.g., sugars, amino acids, ions, or vitamins). Additionally, species interaction networks^166^ may provide insights into the “pantryome” relationship, explaining which species collaborate (e.g., co-occurring species) or compete for resources (e.g., species with negative associations). Advanced methods, such as metabolic modeling of entire microbial communities, could further enhance these analyses.^167^ While promising, such approaches remain challenging due to the complexity of modeling diverse microbial species. For example, interspecies interactions present significant challenges,^168^ and the need for extensive prior knowledge and parameterization further complicates the process.^169^

Finally, predictive bioinformatics can also extend to environmental adaptations. For example, based on taxonomic or functional data, tools can estimate metrics like the MAPI index.^85^ salinity tolerance^170^ and pH preference^171^ of microbial species or communities. Such approaches have also been applied to predict environmental redox potential from microbial community compositions, linking carbon oxidation states with redox conditions and shift in redox potential serving as important indicators of mucosal health.^172,173^ Together, these predictions will enhance our understanding of individual microbiome responses to new microbiome interventions, providing deeper insights into the underlying pathways and mechanisms that influence both microbiome and host health. The recent international consensus statement on microbiome testing in clinical practice will further help shape future directions and pave the way for evidence-based development and use of microbiome testing in clinical investigations.^174^

## 7. Conclusions and outlook

In healthy adults, the gut microbiome is fully developed to maintain balance and promote its own survival against environmental stressors. Recent studies reveal the remarkable adaptability of gut microbial ecosystems, emphasizing their ability to preserve balance through interrelated network structures with microbial micronutrient sharing, gut redox handling and keystone species as possible key elements. Understanding these mechanisms across all life stages, from infancy to adulthood to old age, while accounting for the variability in microbiome profiles shaped by factors such as genetics, diet, lifestyle, and environmental exposures will likely enable the design of better-tolerated and more precise interventions that holistically target functional microbiome networks rather than individual species or strains.

Current pre- and probiotics are widely available on the market and are recognized for their various health benefits, primarily through their role in modulating the gut microbiome. Despite the fact that they are clinically well established for a variety of health benefits, they often exhibit high variability in clinical outcomes due to differences in host physiology, diets, individual microbiome compositions, and other environmental factors. In addition, the living nature of probiotics and the high doses required for traditional prebiotics often limits their applicability in new product formats and ingredient combinations. Thus, there is a need for novel gut health ingredients to provide consistent effects across different individuals, regardless of interindividual microbiome variability.

**Figure 5.**
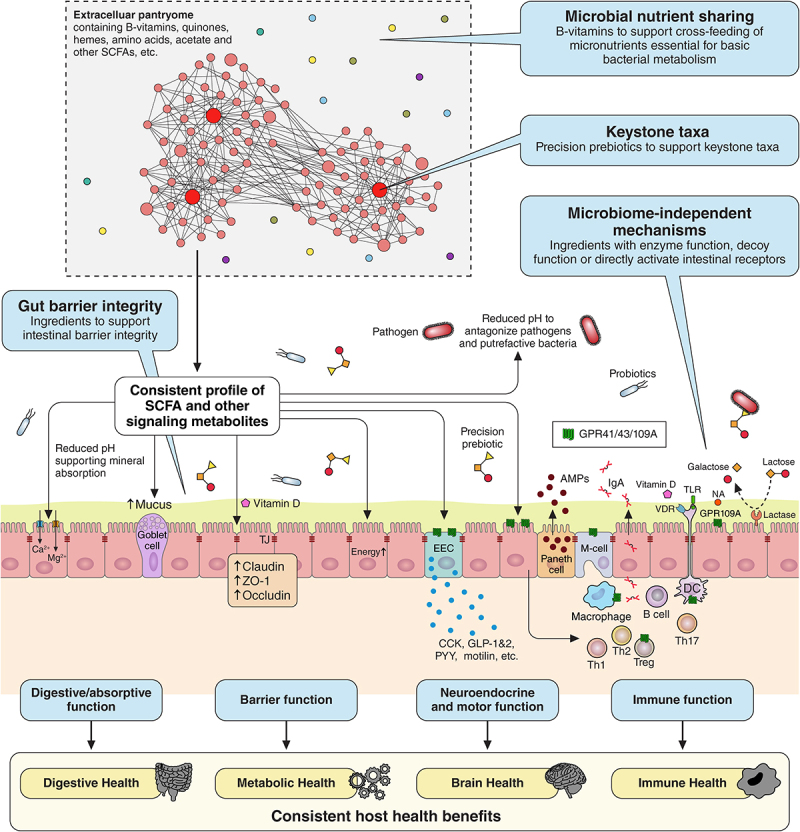
Proposed key pillars of next-generation precision formulations targeting health from the gut. Next-generation ingredient combinations could be devoloped based on the support of (1) microbial micronutrient sharing via the supply of colon-targeted vitamins. This ensures adequate levels of micronutrients essential for bacterial metabolism promoting growth and stabilizing fluctuations in the gut’s luminal redox balance; (2) keystone taxa through carbohydrate-based precision prebiotics for metabolic trophic support 3) the intestinal barrier which plays a fundamental role in host health and disease and is tightly linked to gut microbial health. This can be accomplished through eg. colon-delivered short chain fatty acids (SCFAs) or indirectly through the support of (1) and (2) resulting in a consistent increase SCFA production; and (4) population-specific microbiome-independent mechanisms such as the supply of digestive enzymes or vitamins promoting e.g. immune functioning. Different building blocks could be adapted to target a specific health condition or an individual’s life stage so that complementary modes of action can overcome the limitations of current single-biotic solutions and generate consistent host health benefit.

Here, we highlighted some of the recent scientific insights into the importance of microbial nutrient sharing, gut redox balance, keystone species and the gut barrier for supporting a diverse and fully functional microbial ecosystem and how this can impact host health. We propose that the supply of microbial micronutrients as well as precision prebiotics as part of precisely tailored ingredient combinations may efficiently support and balance microbial ecosystems including keystone taxa to allow a more robust and reliable approach across different subjects and environmental backgrounds (Figure 5). While the inherent interindividual variability of the human gut microbiome (often described as unique ´microbiome fingerprint´) may pose challenges for developing interventions with consistent effects across populations, we speculate that precision prebiotics may address this by being accessible to only a select group of bacteria, thus, reducing competition and potentially leading to a more predictable and consistent prebiotic response across different populations.^143^ As mentioned earlier, recent studies on HMOs or complex fiber structures such as pectin-derived polysaccharides, beta glucans or synthetic glycans support this hypothesis,^148,151–153^ although further research is needed to better demonstrate that targeting a limited set of specific (keystone) taxa could optimize essential metabolic pathways and overall microbial ecosystem performance. When combined with microbial micronutrients such as colon-delivered vitamins to support wider bacterial metabolism and overall ecosystem dynamics as well as to protect from oxidative stress, this approach could form the foundation for a more consistent modulation of the gut microbiome. While additional research is clearly necessary to explore these interactions in depth, early studies suggest significant synergies between microbially produced B-vitamins and classical fermentative metabolic responses.^92,156^ It is important to consider that microbial nutrient requirements are likely encoded in the genome and may remain relatively constant unlike the dynamic metabolic fermentative interactions among community members, which may change over time depending on background diets.^47–49^

Novel precision formulations designed to allow robust and consistent effects across individuals may also lever a diverse range of additional building blocks including 1) colon-targeted SCFAs to support the gut barrier and gut neuroendocrine functions^115^ 2) digestive enzymes to promote microbiome-independent mechanisms, such as macronutrient breakdown and digestions (e.g. lactase for patients with lactose intolerance or IBS)^175^ 3) postbiotics which are known to co-aggregate with pathogens through various mechanisms to help reducing the colonization of harmful microbes^12^ 4) probiotics which are well documented for their wide ranging health benefits^10^ and 5) conventional vitamins and minerals (e.g. vitamin D and zinc) which are established for their immune benefits^176^ (Figure 5). The different building blocks could be chosen to target a specific health condition by evaluating how the key pillars of a specific health/disease phenotype are affected and identifying the necessary ingredients to restore and rebalance them. While the ingredients will likely vary depending on the health condition and an individual’s life stage, their complementary modes of action may overcome the limitations of current single-biotic solutions. This strategy would enable an approach of ´mass´ personalization, offering adapted concepts for particular population groups – a more feasible approach than fully individualized solutions, which have struggled to succeed in the market due to the need for expensive and time-consuming technologies to measure microbiomes, blood, breath, and other factors at an individual level.
